# Impact of Olive Saplings and Organic Amendments on Soil Microbial Communities and Effects of Mineral Fertilization

**DOI:** 10.3389/fmicb.2021.653027

**Published:** 2021-06-01

**Authors:** Miquel Llimós, Guillem Segarra, Marc Sancho-Adamson, M. Isabel Trillas, Joan Romanyà

**Affiliations:** ^1^Section Environmental Health and Soil Science, Department of Biology, Health and Environment, Faculty of Pharmacy and Food Sciences, Universitat de Barcelona, Barcelona, Spain; ^2^Section Plant Physiology, Department of Evolutionary Biology, Ecology and Environmental Sciences, Faculty of Biology, Universitat de Barcelona, Barcelona, Spain

**Keywords:** compost, DNA high-throughput sequencing, microbiome, soil fertility, arbuscular mycorrhizal, MicroResp^TM^, N_2_-fixing

## Abstract

Plant communities and fertilization may have an impact on soil microbiome. Most commercial olive trees are minerally fertilized, while this practice is being replaced by the use of organic amendments. Organic amendments can both fertilize and promote plant growth-promoting organisms. Our aims were (i) to describe the changes in soil bacterial and fungal communities induced by the presence of young olive trees and their interaction with organic amendments and (ii) to compare the effects of mineral and organic fertilization. We set up two parallel experiments in pots using a previously homogenized soil collected from a commercial olive orchard: in the first one, we grew olive saplings in unamended and organically amended soils with two distinct composts and compared these two soils incubated without a plant, while in the second experiment, we comparatively tested the effects of organic and mineral fertilization. OTUs and the relative abundances of bacterial and fungal genera and phyla were analyzed by 16S rRNA and ITS1 gene amplicon using high-throughput sequencing. Basal respiration and substrate-induced respiration were measured by MicroResp^TM^. The effects of the different treatments were analyzed in all phyla and in the 100 most abundant genera. The presence of olive saplings increased substrate-induced respiration and bacterial and fungal richness and diversity. Organic amendments greatly affected both bacterial and fungal phyla and increased bacterial richness while not affecting fungal richness. Mineral fertilization increased the relative abundance of the less metabolically active bacterial phyla (Actinobacteria and Firmicutes), while it reduced the most metabolically active phylum, Bacteroidetes. Mineral fertilization increased the relative abundance of three N_2_-fixing Actinobacteria genera, while organic fertilization only increased one genus of Proteobacteria. In organically and minerally fertilized soils, high basal respiration rates were associated with low fungal diversity. Basidiomycota and Chytridiomycota relative abundances positively correlated with basal respiration and substrate-induced respiration, while Ascomycota correlated negatively. Indeed, the Ascomycota phyla comprised most of the fungal genera decreased by organic amendments. The symbiotrophic phylum Glomeromycota did not correlate with any of the C sources. The relative abundance of this phylum was promoted by the presence of plants but decreased when amending soils with composts.

## Introduction

Microbial communities in agricultural soils may contribute to plant growth and may be affected by farming practices (Emmerling et al., 2001; Pérez-Piqueres et al., 2006). In crops cultivated in soils with a low content of organic matter, such as what is commonly the case with olive tree crops (Romanyà and Rovira, 2011), microbial communities are found to be mainly concentrated around plant roots (Glick, 2018). Organic fertilization can substantially increase soil organic matter in olive grove soils (García-Ruiz and Ochoa, 2012) and, thus, affects soil microbial communities (Landa et al., 2014).

It is estimated that more than 11 million ha of olive trees are cultivated worldwide, notably in Mediterranean countries (Cuevas et al., 2015). Spain is the largest worldwide producer of olive oil with an estimated production of 1.6 × 10^6^ tons of olive oil for the 2018/2019 season (International Olive Oil Council, November 2018). Organic olive farming has largely increased in the world because of its environmental and economic benefits (Pleguezuelo et al., 2018). In Spain, it represents 7.8% of the total olive oil agricultural area (MAPAMA - Ministerio de Agricultura y MedioAmbiente, 2017). The use of compost as fertilizer is promoted by European Regulation 2019/100927. This regulation aims at reducing the demand of mined minerals from third countries by boosting the development of a circular economy [Regulation (EU) 2019/1009]. Moreover, fertilization in organically managed olive tree groves is based on the use of organic amendments, mainly compost. Olive mill waste compost is commonly used in olive tree groves (Muktadirul Bari Chowdhury et al., 2013) due to its environmental and agronomic benefits (Sellami et al., 2007; Aviani et al., 2010; Akratos et al., 2017).

The use of organic amendments affects the chemical and physical properties in soil and may affect soil biology and nutrient availability (Bulluck et al., 2002; Romanyà and Rovira, 2011; Romanyà et al., 2019). Organic amendments have been shown to enhance soil food webs by increasing soil microorganisms and reducing plant-parasitic organisms (Treonis et al., 2010). Organic amendments may have differential effects depending on their quality and the type of soil (Pérez-Piqueres et al., 2006; Saison et al., 2006; Bastida et al., 2008) and may strongly affect soil microbial processes and contribute to soil functions and microbial diversity (Ho et al., 2017; Manjunath et al., 2018).

Large amounts of organic compounds secreted by roots modify microbial populations and activities in soils surrounding the roots, a region defined as the rhizosphere (Giri et al., 2005a). Rhizospheric soils show different properties compared with root-free bulk soils (Berendsen et al., 2012; Bulgarelli et al., 2013) and hold increased numbers and activity of microbes including plant growth-promoting organisms, biological control agents, and pathogens (Giri et al., 2005b; Singh et al., 2011; Fernández et al., 2017). Arbuscular mycorrhizal fungi (AMF) can promote plant growth in low nutrient environments (Nouri et al., 2014). Agricultural soils typically show low AMF diversity (Helgason et al., 1998), particularly in the case of monocultures (Menéndez et al., 2001).

Inorganic fertilizers have been widely used to increase crop yields. However, their effects on microbial biomass and diversity have been studied less. In recent studies, it has been shown that inorganic fertilizers were less favorable to soil microorganisms than organic amendments because they reduced microbial numbers and fungal diversity (Mahanta et al., 2017; Ye et al., 2020). Indeed, it has been reported that microbial diversity increases the quality and fertility of the soil (Garbeva et al., 2004). Soil microorganisms are important to the maintenance of soil function in agriculture because they are involved in key processes such as decomposition of organic matter, toxin removal, soil structure, and the cycling of carbon and nutrients (Trevors and van Elsas, 1997), including symbiotic or asymbiotic N fixation in plant–soil systems (Yoneyama et al., 2017). In symbiotic systems, both mineral and organic fertilization may reduce N_2_ fixation depending on the plant species (Snapp et al., 2017; Romanyà and Casals, 2020). Asymbiotic N-fixing bacteria have been shown to significantly contribute to N cycling, in grasslands (Keuter et al., 2014) and in rice–soil systems, especially with the presence of plants (Bei et al., 2013). Asymbiotic N fixation by free-living bacteria can occur in the rhizosphere or in non-rhizospheric parts of the soils, and the effects of fertilization are less studied in this case. Adding mineral nutrients generally reduces soil organic matter decomposition (Molina-Herrera and Romanyà, 2015; Li et al., 2017), and N-rich microbial metabolites contribute to stabilizing soil organic matter into mineral particle surfaces (Kopittke et al., 2018). Some authors have shown that mineral nutrient additions only decreased bacterial growth, while fungal growth and the use of stabilized C were increased (Silva-Sánchez et al., 2019). Thus, the response of bacterial and fungal communities to soil fertilization appears to be complex and not fully understood.

We hypothesized that growing olive saplings and amending soils with olive mill composts would increase microbial richness and diversity and favor the functioning of soil microorganisms in contrast to mineral fertilization. Specifically, our objectives were (i) to evaluate the contribution of olive saplings and organic amendments to build up the soil microbial community and (ii) to understand how the composition and functioning of this microbial community is affected by mineral and organic fertilization.

## Materials and Methods

### Study Design and Sampling

A greenhouse experiment was developed at the University of Barcelona Torribera Campus (41°27′48.4′′N, 2°12′53.0′′E) employing 2-year-old olive tree clones (*Olea europaea* L.) of the cultivar Picual. In order to homogenize their size, all the plants were pruned down to 7–8 leaves at the start of the experiments. Each sapling was planted in one 10-L pot containing one of the three studied soils used in the experiment. Plants were drip irrigated with water according to demand, with minimal drainage and maintaining soils close to field capacity. Environmental conditions varied in accordance with seasonal changes but were adjusted to a certain extent by automatically controlling the opening and closing of the greenhouse roof in order to avoid extreme temperatures and rainfall. Inside the greenhouse, air temperatures ranged approximately from 6°C (winter) to 30°C (summer) with a relative humidity of 11–59%.

In order to set up the experiments, soil was collected from a commercial olive orchard situated in Abrera, Catalonia, Spain, and amended or not (controls) with two distinct commercial olive mill waste compost. The original soil was a sandy loam (14.5% clay) described as a Calcaric Cambisol (WRB, 2015). Organic C content was 0.86%, C/N ratio was 10.7, and pH was 8.58. For more details (see Romanyà et al., 2019). This soil was sieved through 0.5 cm mesh size, thoroughly homogenized, and mixed with Perlite (Premium Gramoflor from Germany, 2–6 mm) in order to improve aeration. The ratio was 2 soil:1 perlite (v/v). This soil perlite mixture was used to prepare the three different soils of the experiment: unamended soil (SU), soil amended with compost 1 (SC1), and soil amended with compost 2 (SC2). Each compost was separately mixed with soil (4: soil–perlite mix, 1: compost, v/v).

Compost 1 (C1) was composed of 63% wet olive husks, 30% olive leaf waste, and 7% sheep manure, produced in an olive mill in Jaén, Spain, while compost 2 (C2) was composed of 50% wet olive husks, 1% olive leaf waste, and 49% goat manure and straw and was produced in Málaga, Spain. N content was 1.52% in C1 and 1.97% in C2. More details on soil characteristics can be found in Romanyà et al. (2019). Each pot was treated with mineral fertilization (MF+) or not (MF−). An NPK fertilizer (ENTEC Nitrofoska 14–7–17, EuroChem Agro, Barcelona, Spain) was applied at a dose of 142.5 kg N ha^–1^, 31.1 kg P ha^–1^, and 150.45 kg K ha^–1^. No calcium was added in mineral fertilization.

Table 1 describes the setup of treatments in pots. The nine pots without plant were located in the greenhouse and irrigated and maintained free of adventitious plants during the experiments. Soils were sampled 20 months after planting. Plants were grown in 60 pots, 10 per treatment (Table 1). All treatments were randomly distributed on greenhouse benches. The first experiment (objective 1) evaluated the effects of plant (presence or absence of young olive trees) and organic amendments (SU, SC1, SC2) with three replicates and consisted of a total of 18 pots including the 9 pots without plant and 3 randomly selected for MF− treatments with plant for each soil type (SU, SC1, SC2). The second experiment (objective 2) evaluated the effect of mineral fertilization (presence or absence of mineral fertilization) and organic amendments (SU, SC1, SC2) with three replicates, consisting of another group of 18 pots with plant, with 9 pots randomly selected for each MF+ and MF− treatment.

**TABLE 1:** Experimental layout.

**Number of pots**	**Unamended soil (SU)**		**Soil + compost 1 (SC1)**		**Soil + compost 2 (SC2)**	
	**MF−**	**MF+**	**MF−**	**MF+**	**MF−**	**MF+**
Plant+	10	10	10	10	10	10
Plant−	3	0	3	0	3	0

**MF refers to mineral fertilization and plant+ to the presence of olive tree saplings in pots.**

Soil sampling was carried out 20 months after planting. All selected pots were sampled using a 1.4-cm diameter auger. Eight samples were taken from each pot and combined into one bulk sample per pot. Roots were separated from the soils by hand and assumed that soils from pots with plants contained a sample of rhizosphere while soils from pots without plant did not. All bulk soil samples were thoroughly homogenized prior to analyses and were stored in a refrigerator before DNA and MicroResp^TM^ analyses.

### DNA Extraction and Library Construction

Soil DNA was extracted and purified from 1 g of each soil sample using the E.Z.N.A.^TM^ Soil DNA isolation kit following the manufacturer’s protocol. Finally, the quality and quantity of DNA was checked using a NanoDrop (NanoPhotometer P-Class, Implen GmbH, Germany). The amplification and the library construction were performed at MR DNA^1^ (Shallowater, TX, United States). The 16S rRNA gene V4 variable region (for bacterial communities) and the ITS1 gene (for fungal communities) were analyzed using the primers illCUs515F GTGYCAGCMGCCGCGGTAA and new806RB GGACTACNVGGGTWTCTAAT for bacteria and ITS1F-Bt1 CTTGGTCATTTAGAGGAAGTAA and ITS2R GCTGCGTTCTTCATCGATGC for fungi, which have been reported to give improved performance (Walters et al., 2015). Polymerase chain reaction (PCR) consisted of a single-step 30-cycle PCR using the HotStarTaq Plus Master Mix Kit (Qiagen, United States). The PCR conditions were 94°C for 3 min, followed by 30 cycles of 94°C for 30 s, 53°C for 40 s, and 72°C for 1 min, after which a final elongation step at 72°C for 5 min was carried out. After amplification, PCR products were checked in 2% agarose gel to determine the success of amplification and the relative intensity of the bands. Multiple samples were pooled together (e.g., 100 samples) in equal proportions based on their molecular weight and DNA concentrations. Pooled samples were purified using calibrated AMPure XP beads. The pooled and purified PCR product was used to prepare the Illumina TruSeq Nano DNA library.

### Sequencing and Bioinformatics Analysis

Sequencing was performed at MR DNA (see text footnote 1, Shallowater, TX, United States) using the Illumina MiSeq sequencing platform following the manufacturer’s guidelines. Sequence data were processed using a standardized analysis pipeline (Chiodini et al., 2015). In brief, sequences were joined and depleted of barcodes, sequences <150 bp were removed, and sequences with ambiguous base calls were removed. Sequences were denoised, operational taxonomic units (OTUs) generated, and chimeras removed using UCHIME. Operational taxonomic units were defined after the removal of singleton sequences, clustering at 3% divergence (97% similarity) using UCLUST in standard default. Final OTUs were taxonomically classified using BLASTn against a curated database derived from RDPII^2^ and NCBI^3^ databases. The sequence data generated in this study were deposited in the NCBI Sequence Read Archive under BioProject ID PRJNA628525.

### Substrate-Induced Respiration Determination Using MicroResp^TM^

MicroResp^TM^ technology was used to analyze the metabolic response of soil microbiota to different carbon sources (Campbell et al., 2003; Chen et al., 2008). Prior to MicroResp analyses, all soils were dried at room temperature, added to deep 96-well plates, rewetted to 60% field capacity, and incubated for 5 days. Then, 15 selected substrates were added to the deep well plates with the soil samples (triplicate for each sample) to measure substrate-induced respiration (SIR) for 5 h at 30 mg C g^–1^ soil in water for L-cysteine, L-lysine, L-alanine, L-arabinose, trehalose, D-fructose, D-galactose, D-glucose, γ-aminobutyric acid, citric acid, oxalic acid, L-malic acid, or orcinol and at 7.5 mg C g^–1^ soil in water for arginine or *N*-acetylglucosamine. To calculate the SIR, we used the difference between the CO_2_-C (mg) produced in soil with the addition of each substrate to the CO_2_-C produced in the same soil with only water added. Furthermore, the mean of the SIR of the 15 selected substrates was taken as the average SIR. Respiration in the treatment that only received water was taken as a measure of basal respiration.

### Statistical Analysis

Calypso version 8.84 online free package software (Zakrzewski et al., 2017) was used to run principal coordinates analyses (PCoA) using a Bray–Curtis dissimilarity matrix on the OTU data from each of the two experiments. All data were normalized by square root transformation before the statistical analysis. PERMANOVA was performed using Adonis function and a Bray–Curtis dissimilarity matrix and was also calculated using Calypso. Bacterial and fungal richness and Shannon alfa diversity indexes were calculated using Calypso at the OTU level. The effects of treatments of both experiments on the 100 most abundant genera of fungi or bacteria were analyzed by two-way ANOVA for each of the two experiments using Calypso and used the false discovery rate as criteria of significance. Factors were the presence of plant and soil amendments in experiment 1 and mineral fertilization and soil amendments in experiment 2.

Square root-transformed relative abundance of phyla data from each experiment was analyzed using SPSS software (IBM Corp. Released 2013. IBM SPSS Statistics for Windows, Version 21.0. Armonk, NY: IBM Corp.). Two-way ANOVA for each experiment and Tukey’s multiple range test (*P* < 0.05) were used to identify significant differences between soils (SU, SC1, and SC2). Pearson’s product-moment correlation coefficient was performed for each of the two experiments to explore correlations between the fungal and bacterial phyla and the SIR of the different treatments.

## Results

### Identification of Soil Microbial Communities Using DNA High-Throughput Sequencing

The dominant bacterial phylum across all the treatments was Proteobacteria, representing more than 50% of the bacterial sequences from each of the soils (Table 2). The relative abundance of this phylum was not affected by the presence of plants nor by the addition of compost (Table 2). The presence of plants increased the relative abundance of Planctomycetes and Acidobacteria. Adding composts increased the relative abundance of Firmicutes and Bacteroidetes, while the phylum Chloroflexi increased only in the case of SC1. In contrast, the addition of both composts reduced the abundances of Gemmatimonadetes and Planctomycetes, while Thaumarcheota were only reduced in the case of SC1 soils.

**TABLE 2:** Relative abundances (%) and standard errors of the most abundant bacterial phyla in the three soils (SU, unamended soil; SC1, soil amended with compost 1; SC2, soil amended with compost 2), with (plant+) or without (plant−) olive sapling plant.

	**SU**		**SC1**		**SC2**		**ANOVA**		
	**Plant−**	**Plant+**	**Plant−**	**Plant+**	**Plant−**	**Plant+**	**P**	**S**	P*S
Proteobacteria	65.6 ± 7.3	55.6 ± 1.2	61.7 ± 5.1	61.8 ± 1.8	65.0 ± 9.1	59.9 ± 0.7	n.s.	n.s.	n.s.
Actinobacteria	11.2 ± 3.0	15.0 ± 1.2	12.6 ± 5.4	10.1 ± 0.9	8.3 ± 2.2	10.7 ± 0.8	n.s.	n.s.	n.s.
Firmicutes	3.6 ± 1.2	5.4 ± 0.1	5.2 ± 0.6	7.2 ± 1.1	7.0 ± 1.9	8.4 ± 0.3	n.s.	0.043	n.s.
Bacteroidetes	2.9 ± 0.6	3.9 ± 0.6	5.5 ± 0.5	5.7 ± 0.5	3.7 ± 1.2	5.3 ± 0.5	n.s.	0.043	n.s.
Chloroflexi	2.4 ± 0.8	3.3 ± 0.2	6.7 ± 1.3	5.0 ± 0.7	3.8 ± 1.1	2.8 ± 0.3	n.s.	0.008	n.s.
Gemmatimodetes	3.9 ± 0.9	4.1 ± 0.1	1.9 ± 0.2	2.7 ± 0.2	3.4 ± 1.0	3.3 ± 0.2	n.s.	0.024	n.s.
Planctomycetes	2.0 ± 0.5	3.5 ± 0.1	1.4 ± 0.2	1.7 ± 0.1	1.9 ± 0.5	2.4 ± 0.2	0.024	0.021	n.s.
Acidobacteria	2.2 ± 0.5	3.4 ± 0.2	1.5 ± 0.2	2.4 ± 0.2	1.9 ± 0.4	2.9 ± 0.7	0.005	n.s.	n.s.
Thaumarcheota	1.6 ± 0.6	1.3 ± 0.1	0.5 ± 0.1	0.5 ± 0.0	1.7 ± 0.5	1.0 ± 0.0	n.s.	0.007	n.s.
Others	3.8 ± 0.6	4.7 ± 0.3	3.4 ± 0.3	2.9 ± 0.3	3.4 ± 1.0	3.3 ± 0.2	n.s.	n.s.	n.s.

*Significance of two-way ANOVA is also shown.*

P, plant effect; S, soil effect; P*S, interaction between plant and soils; n.s., not significant.

**FIGURE 1:**
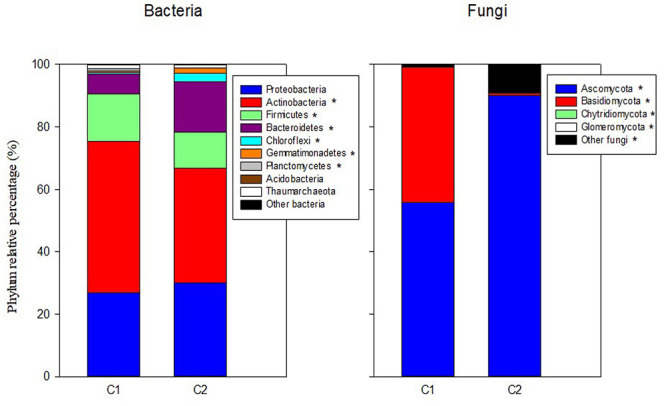
Percentages of the relative abundance of dominant fungal and bacteria phyla in the two original composts (C1, C2) prior to soil application. Asterisks in the legend indicate differences in the relative abundance for each phylum between the two composts.

In the mineral fertilization–soil experiment, MF decreased the abundances of Proteobacteria and Bacteroidetes, while it increased the abundance of Actinobacteria, Firmicutes, and Thaumarchaeota (Table 3). In this experiment, Planctomycetes increased only in SC1 soils. Planctomycetes were virtually absent from C2 compost (Figure 1). C1 had more Actinobacteria (48.41%) and Firmicutes (15.17%) compared with C2, and C2 had higher relative abundance of Bacteroidetes (16.23%), Chloroflexi (2.78%), and Gemmatimonadetes (1.55%) compared with C1.

**TABLE 3:** Relative abundances (%) and standard errors of the most abundant bacterial phyla in the three soils (SU, unamended soil; SC1, soil amended with compost 1; SC2, soil amended with compost 2), with (MF+) or without (MF−) mineral fertilization.

	**SU**		**SC1**		**SC2**		**ANOVA**		
	**MF−**	**MF+**	**MF−**	**MF+**	**MF−**	**MF+**	**MF**	**S**	MF*S
Proteobacteria	55.6 ± 1.2	51.6 ± 2.8	61.8 ± 1.8	53.9 ± 1.2	60.0 ± 0.7	50.4 ± 3.7	0.002	n.s.	n.s.
Actinobacteria	15.0 ± 1.2	19.0 ± 3.0	10.1 ± 1.0	14.4 ± 0.8	10.7 ± 0.8	17.2 ± 1.1	0.001	0.022	n.s.
Firmicutes	5.4 ± 0.1	8.8 ± 0.8	7.2 ± 1.1	8.5 ± 0.6	8.4 ± 0.3	11.8 ± 1.5	0.002	0.009	n.s.
Bacteroidetes	3.9 ± 0.6	2.8 ± 0.2	5.7 ± 0.5	4.2 ± 0.2	5.3 ± 0.5	4.4 ± 0.1	0.005	0.002	n.s.
Chloroflexi	3.3 ± 0.2	3.5 ± 0.4	5.0 ± 0.7	5.3 ± 0.4	2.8 ± 0.3	4.3 ± 0.4	n.s.	0.002	n.s.
Gemmatimodetes	4.1 ± 0.1	3.6 ± 0.5	2.7 ± 0.2	3.6 ± 0.4	3.3 ± 0.2	2.9 ± 0.1	n.s.	0.042	n.s.
Planctomycetes	3.5 ± 0.1	3.1 ± 0.1	1.7 ± 0.1	2.8 ± 0.4	2.4 ± 0.2	2.3 ± 0.2	n.s.	< 0.001	0.002
Acidobacteria	3.4 ± 0.2	2.8 ± 0.2	2.4 ± 0.2	2.9 ± 0.2	2.9 ± 0.7	1.9 ± 0.1	n.s.	n.s.	n.s.
Thaumarcheota	1.3 ± 0.1	1.5 ± 0.0	0.5 ± 0.0	1.1 ± 0.2	1.0 ± 0.0	1.4 ± 0.3	0.003	0.003	n.s.
Others	4.7 ± 0.3	3.4 ± 0.4	3.0 ± 0.3	3.5 ± 0.6	3.3 ± 0.2	3.5 ± 0.3	n.s.	n.s.	n.s.

*Significance of two-way ANOVA is also shown.*

MF, mineral fertilization effect; S, soil effect; MF*S, interaction between mineral fertilization and soils.

Ascomycota was the dominant fungal phylum showing relative abundances higher than 40% (Tables 4, 5). The relative abundance of this phylum decreased with the presence of plants only in SU and SC2 soils (Table 4). In contrast, the presence of plants increased the abundance of Basidiomycota in SU and SC2 soils. In contrast with C1, the presence of Basidiomycota in C2 was very low (Figure 1). The relative abundances of fungal phyla in composts showed that C1 had 55.88% of Ascomycota and 43.26% of Basidiomycota, while C2 had 90.06% of Ascomycota with low relative abundance of the other phyla including Basidiomycota (Figure 1). Glomeromycota were virtually absent in both composts, and their relative abundance increased with the presence of plants in all soils and decreased when adding composts (SC1 and SC2 soils). Chytridiomycota showed a large increase only in SC1 soils. MF did not show any effect on fungal phyla (Table 5).

**TABLE 4:** Relative abundances (%) and standard errors of the most abundant fungal phyla in the three soils (SU, unamended soil; SC1, soil amended with compost 1; SC2, soil amended with compost 2), with (plant+) or without (plant−) olive sapling plant.

	**SU**		**SC1**		**SC2**		**ANOVA**		
	**plant−**	**Plant+**	**plant−**	**Plant+**	**plant−**	**Plant+**	**P**	S	P*S
Ascomycota	69.8 ± 5.4	54.8 ± 2.9	39.1 ± 3.9	42.7 ± 1.7	83.9 ± 6.3	50.5 ± 7.7	0.006	< 0.001	0.013
Basidiomycota	11.5 ± 3.0	20.2 ± 3.4	41.3 ± 4.4	31.0 ± 4.4	12.1 ± 5.5	33.3 ± 9.6	n.s.	0.008	0.039
Chytridiomycota	10.7 ± 4.9	3.1 ± 0.7	17.6 ± 7.5	20.4 ± 5.4	1.0 ± 0.2	7.5 ± 3.6	n.s.	0.006	n.s.
Glomeromycota	1.9 ± 0.6	9.1 ± 1.9	1.5 ± 0.2	3.6 ± 0.5	1.0 ± 0.4	2.8 ± 0.5	< 0.001	0.003	0.05
Others	6.1 ± 0.5	12.8 ± 4.8	0.6 ± 0.1	2.3 ± 0.9	2.0 ± 0.6	6.0 ± 1.8	0.014	0.001	n.s.

*Significance of two-way ANOVA is also shown.*

P, plant effect; S, soil effect; P*S, interaction between plant and soils.

**TABLE 5:** Relative abundances (%) and standard errors of the most abundant fungal phyla in the three soils (SU, unamended soil; SC1, soil amended with compost 1; SC2, soil amended with compost 2), with (MF+) or without (MF−) mineral fertilization.

	**SU**		**SC1**		**SC2**		**ANOVA**		
	**MF−**	**MF+**	**MF−**	**MF+**	**MF−**	**MF+**	**MF**	**Soil**	MF*S
Ascomycota	54.8 ± 2.9	51.3 ± 4.2	42.7 ± 1.7	56.5 ± 11.5	50.5 ± 7.7	69.3 ± 10.6	n.s.	n.s.	n.s.
Basidiomycota	20.2 ± 3.4	25.4 ± 8.4	31.0 ± 4.4	29.7 ± 10.2	33.3 ± 9.6	22.6 ± 11.5	n.s.	n.s.	n.s.
Chytridiomycota	3.1 ± 0.7	6.3 ± 0.7	20.4 ± 5.4	10.0 ± 2.0	7.5 ± 3.6	5.2 ± 2.9	n.s.	0.009	n.s.
Glomeromycota	9.1 ± 1.9	8.3 ± 3.8	3.6 ± 0.5	2.6 ± 0.7	2.8 ± 0.5	1.1 ± 0.1	n.s.	0.001	n.s.
Others	12.8 ± 4.8	8.8 ± 2.2	2.3 ± 0.9	1.9 ± 0.4	6.0 ± 1.8	1.8 ± 0.4	n.s.	0.002	n.s.

*Significance of two-way ANOVA is also shown.*

MF, mineral fertilization effect; S, soil effect; MF*S, interaction between mineral fertilization and soils.

**FIGURE 2:**
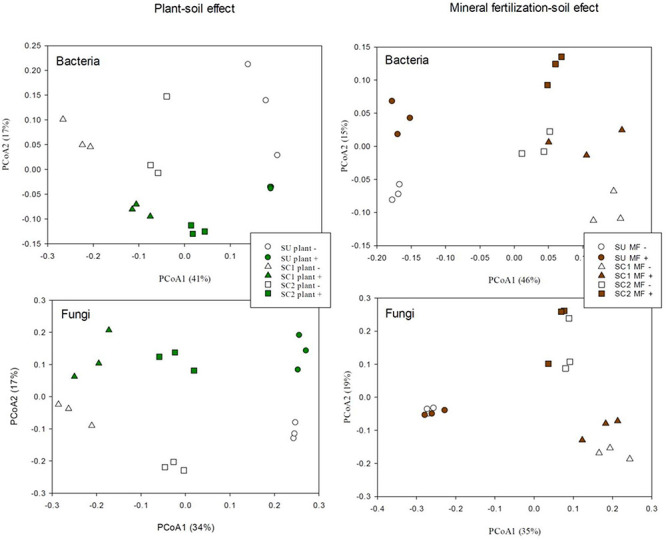
Principal coordinate analysis (PCoA) of bacterial and fungal operational taxonomic units (OTUs) in the three different used soils (S, SC1, SC2) combined with the effects of the presence of plant and the effects of used soils (S, SC1, SC2) combined with mineral fertilization in the presence of plants.

PCoA of the bacterial communities (Figure 2) clearly discriminated in the *X*-axis (41%) the SU from the two compost-amended soils. The *Y*-axis (17%) discriminated between soils with and without plant (Figure 2). In relation to the effects of fertilization in bacterial communities (Figure 2), the PCoA discriminated between the SU and the two amended soils as can be seen in the *X*-axis (46%), while the discrimination between the fertilized and non-fertilized treatments is shown in the *Y*-axis (15%) in which a similar pattern was observed for all soils (SU, SC1, SC2). PERMANOVA analysis revealed significant differences among substrates (SU, SC1, and SC2; *P* < 0.001) and the presence of plant (*P* < 0.05). However, in the mineral fertilization–soil experiment, significant differences were only found between substrates (*P* < 0.001).

PCoA of the fungal communities from the soils with and without plant (Figure 2) discriminated in the *X*-axis (34%) between the different soils (SU, SC1, SC2), while the *Y*-axis (17%) discriminated between soils with and without plant. In relation to the effect of mineral fertilization in bacterial communities (Figure 2), the PCoA discriminated between the unamended soils and the two amended soils by combining the *X*-axis (35%) and the *Y*-axis (19%). PCoA did not show any discrimination effect due to fertilization for the unamended soils (SU). PERMANOVA analysis revealed significant differences among substrates (*P* < 0.001) and the presence of plant (*P* = 0.013). However, significant differences were only found for substrates (*P* < 0.001) in the mineral fertilization–soil experiment.

### Diversity of Bacterial and Fungal Communities

**FIGURE 3:**
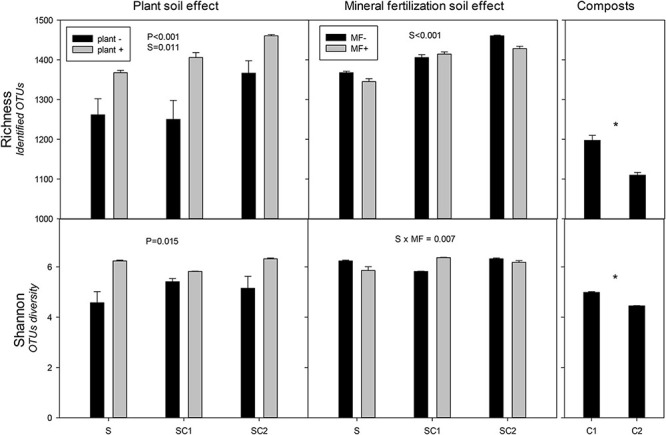
Richness and Shannon index for the identified operational taxonomic units (OTUs) of bacteria in the different soils (S, SC1, SC2) with (plant+) and without plant and in minerally fertilized (MF+) and unfertilized (MF−) soils (S, SC1, SC2). Data on the original composts (C1 and C2) are also shown. Data represent the means and standard errors of three replicates for each sample. Significant factors of ANOVA are indicated. P, plant effect; S, soil effect; MF, mineral fertilization; S*MF, interaction of soils and mineral fertilization. Asterisk indicates significant differences between the composts.

Significant effects of plant and soils on the richness of bacterial communities were observed, with the highest values obtained in the presence of plant in soils amended with composts (Figure 3). The highest value was found in SC2 soils with plants. The Shannon index revealed a clear effect of plant on bacterial communities regardless of the effect of the soils, in which the lowest value was obtained for SU without plant (Figure 3). There was a significant effect of organic amendments on the richness index of the bacterial communities in the soils, with the highest values corresponding to SC2 (Figure 3). Moreover, the Shannon index showed an interaction between soils and mineral fertilization (Figure 3). Composts showed less richness compared with soils and C1 had more richness than C2. Similarly, in Shannon diversity index, C1 showed higher results compared with C2.

**FIGURE 4:**
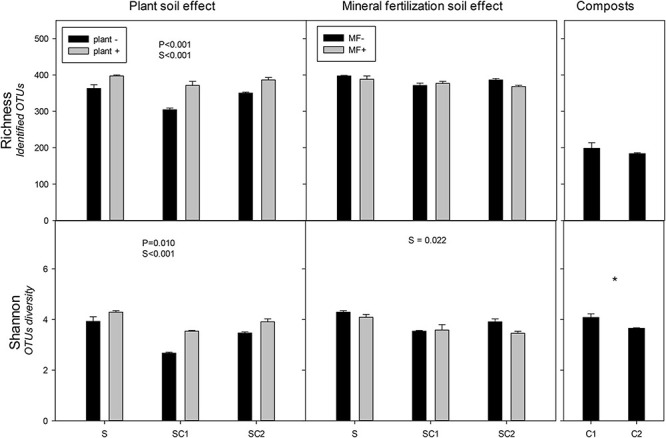
Richness and Shannon index for the identified operational taxonomic units (OTUs) of fungi in the different soils (S, SC1, SC2) with (plant+) and without plant and in minerally fertilized (MF+) and unfertilized (MF−) soils (S, SC1, SC2). Data on the original composts (C1 and C2) are also shown. Data represent the means and standard errors of three replicates for each sample. Significant factors of ANOVA are indicated. P, plant effect; S, soil effect; MF, mineral fertilization; S*MF, interaction of soils and mineral fertilization. Asterisk indicates significant differences between the composts.

The presence of plants increased the richness and Shannon indexes of fungal communities (Figure 4). Contrasting with the values obtained for bacteria, the highest values were found in SU in the presence of plant and the lowest in SC1 without plant. Soil amended with compost showed lower values than unamended soils. The lowest values of both richness and Shannon were observed in SC1 soils. The effect of mineral fertilization did not influence the richness and Shannon index of the fungal communities (Figure 4). In contrast with bacteria, the highest Shannon index value occurred in unfertilized soils (SU), while no significant effects were found for richness. However, in fertilized soils, fungal richness correlated negatively (*r* = −0.491, *P* = 0.039) with bacterial richness. Composts showed less richness compared with the soils. Shannon diversity was higher in C1 than in C2 compost.

### Substrate-Induced Respiration

**FIGURE 5:**
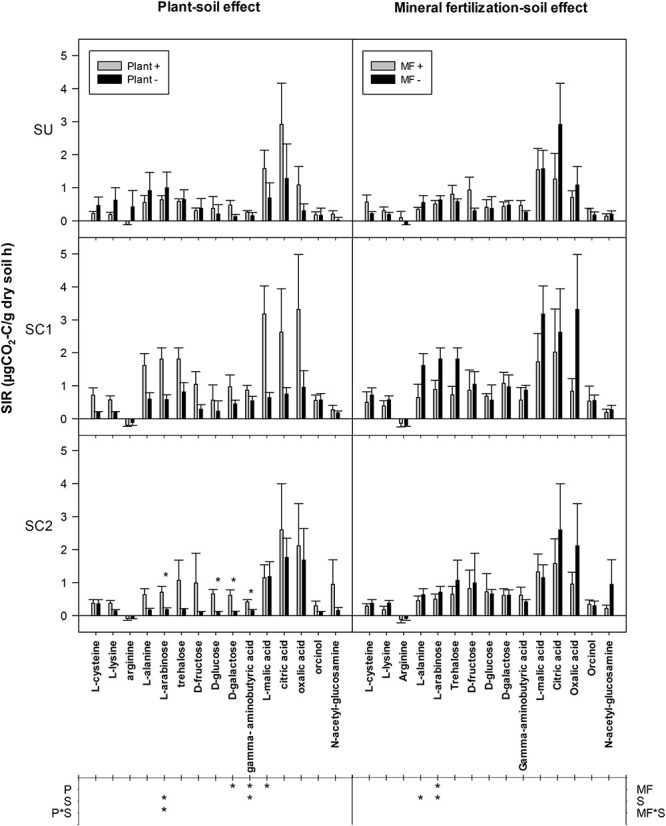
Mean substrate-induced respiration (SIR) rates in response to the addition of 15 C substrates to soils from different treatments. The effects of the different soils (S, SC1, SC2) with (plant+) and without plant (plant-) are shown in the left column (plant–soil effect), while the effects of soils (S, SC1, SC2) and mineral fertilization (MF+, MF−) are shown on the right (mineral fertilization–soil effect). Asterisks in the main plot indicate significance of the plant effects within each soil. The lower table indicates ANOVA significances with an asterisk for factors and interaction in each experiment. P refers to the plant effect, S to the soil effect (S), and P*S to the interaction between plant and soil. Error bars indicate standard error of the mean (*n* = 3).

A significant increase of D-galactose, γ-aminobutyric acid, and L-malic acid SIR was observed in all soils due to the presence of plants (Figure 5). This effect was also observed in the case of L-arabinose in soils amended with compost but not in unamended soils (SU). SC1 soil showed an increased SIR for L-arabinose and γ-aminobutyric acid compared with the other soils. By looking at each individual soil, SIR increased in some cases due to the presence of plants, but only in amended soils: SIR increased in SC1 soils for the substrates L-lysine, L-arabinose, and L-malic acid and in SC2 soils for the substrates L-arabinose, D-glucose, D-galactose, and γ-aminobutyric acid. On the other hand, mineral fertilization did not show any effect on SIR in any of the soils with the exception of a decrease for L-arabinose. SC1 soils showed increased SIR for L-alanine and L-arabinose compared with SU and SC2 soils. Finally, the average SIR for the 15 substrates showed a plant effect for all soils, significantly increasing SIR from 0.46 to 0.94 μg CO_2_-C g^–1^ soil h^–1^ in the presence of plant. Basal respiration rates ranged between 0.13 and 0.79 μg CO_2_-C g^–1^ soil h^–1^ and increased following the addition of different C substrates except for arginine, whose values decreased.

**FIGURE 6:**
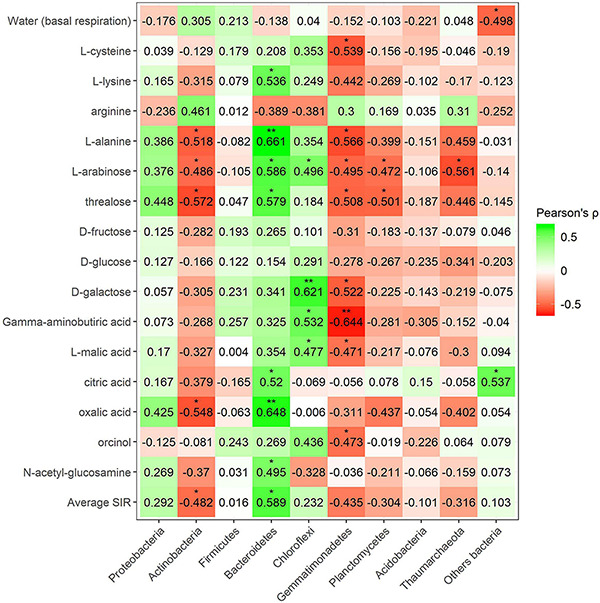
Heatmap of Pearson’s correlation coefficient (*r*) between the most abundant bacterial phyla (plant–soil effect) and the SIR results of the different carbon sources. Heatmap colors represent the Pearson correlations ranges, from red (negative correlation), passing through white (no correlation) to green (positive correlation). Significant results are indicated by an asterisk.

**FIGURE 7:**
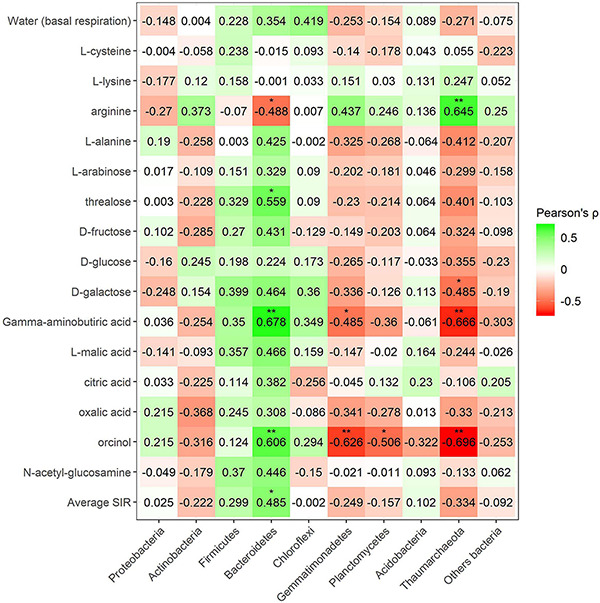
Heatmap of Pearson’s correlation coefficient (*r*) between the most abundant fungal phyla (plant–soil effect) and the SIR results of the different carbon sources. Heatmap colors represent the Pearson correlations ranges, from red (negative correlation), passing through white (no correlation) to green (positive correlation). Significant results are indicated by an asterisk.

**FIGURE 8:**
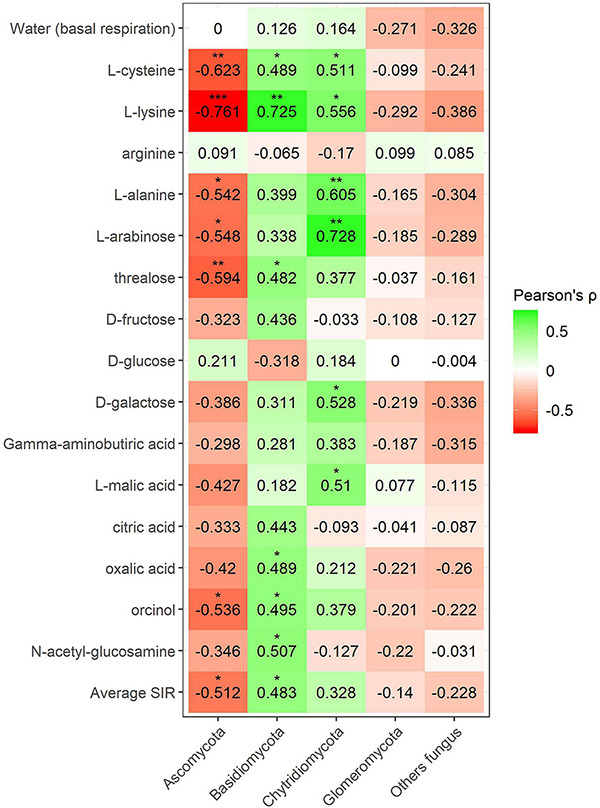
Heatmap of Pearson’s correlation coefficient (*r*) between the most abundant fungal phyla (mineral fertilization–soil effect) and the SIR results of the different carbon sources. Heatmap colors represent the Pearson correlations ranges, from red (negative correlation), passing through white (no correlation) to green (positive correlation). Significant results are indicated by an asterisk.

Pearson’s correlation coefficient obtained from SIR and the relative abundances of the bacterial groups in different soils with and without plant showed three significant positive correlations (trehalose, γ-aminobutyric acid, and orcinol) and one significant negative correlation (arginine) for the phylum Bacteroidetes, out of a total of 15 carbon sources (Figure 6). Gemmatimonadetes showed two negative correlations (γ-aminobutyric acid and orcinol). Planctomycetes showed a negative correlation with orcinol, and finally, Thaumarchaeota showed three positive correlations with galactose, γ-aminobutyric acid, and orcinol. Fungal phyla data from the plant–soil effect experiment showed four negative correlations (trehalose, galactose, γ-aminobutyric acid, and orcinol) for Ascomycota, while Basidiomycota showed another four positive correlations (trehalose, fructose, γ-aminobutyric acid, and *N*-acetylglucosamine). Chytridiomycota also showed four positive correlations with carbon sources L-arabinose, trehalose, γ-aminobutyric acid, and orcinol. No significant correlations were obtained for Glomeromycota or other fungal phyla (Figures 7, 8). While Basidiomycota and Chytridiomycota showed positive relationships with basal respiration, Ascomycota showed a negative relationship. Indeed, Shannon index of fungal OTU relative abundances showed a marginally significant negative correlation with basal respiration (*r* = −0.467, *P* = 0.05). In contrast, neither bacterial phyla abundances nor bacterial OTU’s Shannon index was shown to be related to basal respiration.

**FIGURE 9:**
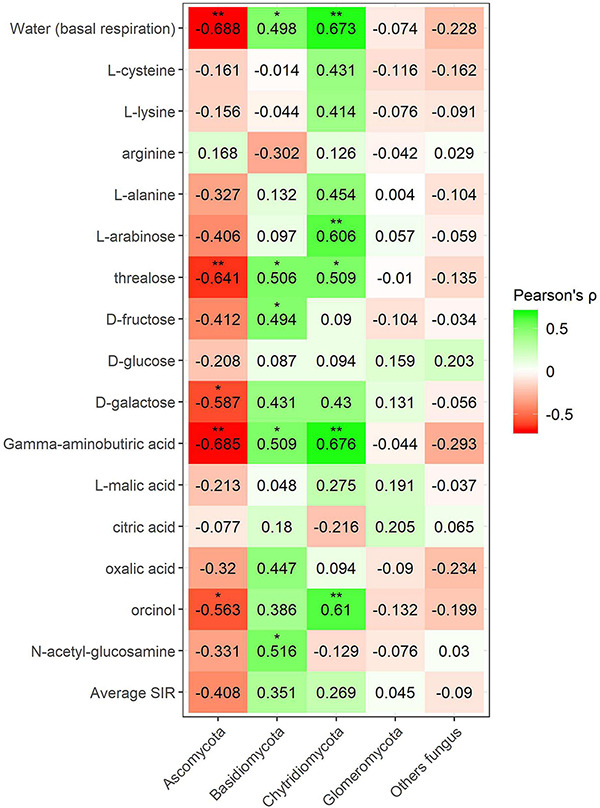
Heatmap of Pearson’s correlation coefficient (*r*) between the most abundant bacterial phyla (mineral fertilization–soil effect) and the SIR results of the different carbon sources. Heatmap colors represent the Pearson correlations ranges, from red (negative correlation), passing through white (no correlation) to green (positive correlation). Significant results are indicated by an asterisk.

Pearson’s coefficient in minerally fertilized soils showed seven positive correlations (lysine, L-alanine, arabinose, trehalose, citric acid, oxalic acid, and *N*-acetylglucosamine) for the phylum Bacteroidetes (Figure 9). Positive correlations for this phylum included amino acids, sugars, and organic acids. SIR also showed four positive correlations with Chloroflexi bacteria (L-arabinose, D-galactose, γ-aminobutyric acid, and L-malic acid). No amino acid correlations were found in this case. Many negative correlations with bacterial phyla were observed in minerally fertilized soils. The relative abundance of Gemmatimonadetes showed as many as eight negative correlations (L-cysteine, L-alanine, L-arabinose, trehalose, galactose, γ-aminobutyric acid, L-malic acid, and orcinol) out of 15 substrates. Actinobacteria also negatively correlated with amino acids, sugars, and organic acids (L-alanine, arabinose, trehalose, oxalic acid). The relative abundance of Planctomycetes and Thaumarcheota only presented negative correlations with certain sugars: arabinose and trehalose in the case of Planctomycetes and arabinose in the case of Thaumarchaeota.

Fungal phyla correlations in minerally fertilized soils, similar to what was observed in soils with and without plants, showed positive relationships with SIR in the case of Basidiomycota and Chytridiomycota (Figure 8). Basidiomycota relative abundance correlated with L-cysteine, L-lysine, trehalose, oxalic acid, orcinol and *N*-acetylglucosamine, and Chytridiomycota correlated with L-cysteine, L-lysine, L-alanine, L-arabinose, D-galactose, and L-malic acid. Unlike in soils with and without plants, these positive correlations included amino acids, while they did not relate to γ-aminobutyric acid. The phylum Ascomycota negatively correlated with L-cysteine, L-lysine, L-alanine, L-arabinose, trehalose, and orcinol. In this case, no correlations were observed for the phylum Glomeromycota nor with basal respiration.

### Changes in Relative Abundances of the Most Abundant Bacterial and Fungal Genera

Significant changes of the 100 most abundant bacterial and fungal genera of the effects of mineral fertilization–soil and of plant–soil experiments are shown in Tables 6, 7. The soil effects refer to the mineral fertilization–soil experiment. The number of genera showing significant effects to each one of the two factors and interactions of the second experiment (soil, mineral fertilization) was counted as well as the number of genera affected by the presence of plant and interactions of the first experiment. In Tables 6, 7, it is also shown that the number of genera increased by each treatment. The difference between the first and third column within each factor gives the number of decreased genera. Bacterial genera with N-fixing capacity have been counted separately. Fungal genera have been classified by trophic modes according to the FUNGuild database (Nguyen et al., 2016). The complete list of genera showing significant changes can be found in the Supplementary Materials.

**TABLE 6:** Number of genera from the 100 most abundant genera of bacteria affected by soil type (compost addition), mineral fertilization, and presence of plant (olive saplings).

**Number of genera affected by:**	**Soil**			**Mineral fertilization**			**Plant**		
	**Soil**	**SMF**	**Incr.**	**MF+**	**MFS**	**Incr.**	**Plant**	**PS**	**Incr.**
**Proteobacteria**									
No N_2_ fixing	26	7	23	7	2	2	17	2	14
N_2_ fixing	2	1	1	3	3	1	5	1	5
**Actinobacteria**									
No N_2_ fixing	13	5	11	2	2	2	6	5	6
N_2_ fixing	3	1	2	3	1	3	3	0	3
**Firmicutes**									
No N_2_ fixing	4	2	3	2	1	1	1	0	1
**Bacteroidetes**									
No N_2_ fixing	3	1	3	1	1	0	1	0	1
**Chloroflexibacteria**									
No N_2_ fixing	4	4	2	2	1	2			
**Planctomycetes**									
No N_2_ fixing	2	1	1	1	0	1	2	1	2
**Acidobacteria**									
No N_2_ fixing							1	0	1
Total	57	22	46	21	11	12	36	5	33

**Incr. refers to the number of affected genera that are increased by adding compost (soil), mineral fertilization, or presence of plant. The number of significant interactions between factors are also indicated. The effects of soil were taken from the soil mineral fertilization experiment. Within each phylum, bacteria have been classified according to their capacity to fix atmospheric N_2_ (N_2_ fixing, no N_2_ fixing).**

**TABLE 7:** Number of genera form the 100 most abundant genera of fungi affected by soil type (compost addition), mineral fertilization, and presence of plant (olive saplings).

**Number of genera affected by**	**Soil**			**Mineral fertilization**			**Plant**		
**Phyla and trophic mode**	**Soil**	**SMF**	**Incr.**	**MF+**	**MFS**	**Incr.**	**Plant**	**PS**	**Incr.**
**Ascomycota**									
Patotroph	1		0				1	1	0
Patotroph–saprotroph	1		0						
Pathotroph–saprotroph–symbiotroph	2		0				2	1	0
Pathotroph–symbiotroph	2	1	1	1	1	1	2	1	1
Saprotroph	17	2	9	2	2	1	7	5	5
Saprotroph–symbiotroph	1		1						
Symbiotroph	2		0						
Unclassified	13	1	5				3	2	3
**Basidiomycota**									
Pathotroph	1		1				1	1	1
Pathotroph–saprotroph	1		1				1	0	0
Pathotroph–saprotroph–symbiotroph	2		0						
Saprotroph	4	1	2	2	2	0	2	2	2
Symbiotroph	4	1	1				3	1	3
Unclassified	1		1				1	0	1
**Chytridiomycota**									
Pathotroph	1		1						
Pathotroph–saprotroph	1		0						
Unclassified	1		1						
**Glomeromicota**									
Symbiotroph	3		1				3	2	3
**Other**									
Pathotroph–saprotroph	1		0				1	0	1
Saprotroph	1		0						
Saprotroph–symbiotroph	1		0						
Symbiotroph	1		0						
Total	62	6	25	5	5	2	27	16	20

**Incr. refers to the number of affected genera that are increased by adding compost (soil), mineral fertilization, or the presence of plant. The number of significant interactions between factors is also indicated. The effects of soil were obtained from the soil mineral fertilization experiment. Within each phylum, fungal genera have been classified in trophic modes according to FUNGuild classification* (Nguyen et al., 2016).*

## Discussion

### Mineral Fertilization–Soil and Plant–Soil Effects

Plants, soil type, and agricultural practices select rhizosphere microbial communities (Pérez-Piqueres et al., 2006; Bulgarelli et al., 2013; Landa et al., 2014; Vega-Avila et al., 2015; Na et al., 2019). Soil microbial communities are in turn sensitive to both organic and mineral fertilization (Ling et al., 2016; Wang et al., 2017; Guo et al., 2019).

In our study, organic amendments affected a greater number of bacterial phyla and bacterial genera than the presence of plants. Moreover, when comparing the two experiments, mineral fertilization affected more bacterial phyla than the presence of plants. These findings contrast with the data obtained from the most abundant genera of bacteria which shows larger numbers affected by plants than by mineral fertilization. In soils with plants, the addition of composts increased bacterial richness and, in some cases, diversity (in minerally fertilized SC1 soils). Richness and diversity of the soil microbiome are important in providing resilience to stress and disease, stability, and high levels of internal nutrient cycling (Elliot and Lynch, 1994). A recent publication (van der Bom et al., 2018) shows an increase of bacterial richness in the presence of animal manure, in accordance with our results, especially those obtained for SC2, which is composed of 49% goat manure. In contrast, bacterial diversity only increased in soils amended with low N compost (C1) and only after mineral fertilization. On the other hand, the addition of compost increased the relative abundance of 46 bacterial genera out of 100, including three N-fixing bacteria (*Frankia*, *Micromonospora*, and *Methylobacterium*), while it decreased two N-fixing genera (*Microvirga* and *Arthrobacter*). Mineral fertilization increased a lower number of bacterial genera (12) including four N-fixing genera (*Streptomyces*, *Frankia*, *Arthrobacter*, and *Devosia*).

The presence of olive plants increased richness and diversity and induced a shift in bacterial phyla, increasing the relative abundance of the phyla Planctomycetes and Acidobacteria and 36 genera, including 8 N-fixing genera.

In the plant–soil context, the addition of compost affected all studied fungal phyla, while in the context of mineral fertilization, adding compost only affected three of the studied phyla (Chytridiomycota, Glomeromycota, and Others). Interestingly, mineral fertilization did not show any effect on fungal richness and diversity and did not increase any fungal phyla, Indeed, MF only increased 2 fungal genera of the 100 most abundant genera (*Thermomyces* and *Thielavia*). In contrast to bacteria, organic amendments did not affect fungal richness and decreased fungal diversity. This may be related to decreases in abundance of more than one third 37 of the most abundant genera, while another 25 genera were largely increased. Genera increased by organic amendments were mostly Ascomycota and Basidiomycota and included two genera of Chytridiomycota. Both the Glomeromycota phylum and genera were reduced by compost additions, except in the case of *Acaulospora* that increased with compost.

It is interesting to point out that in organically and minerally fertilized soils, low fungal diversity was associated with high basal respiration rates. Ascomycota showed a greater number of increased saprotroph genera 10 due to organic amendments. This contrasts with the negative correlation of this phylum with soil basal respiration. Somewhat surprisingly, Basidiomycota showed much lower numbers of increased saprotrophs 3 and a positive correlation with SIR. Glomeromycota was the only phylum showing a single trophic mode (symbiotroph) and coincided with no effects on heterotrophic respiration.

In contrast to organic fertilization, the presence of plants only decreased seven of the most abundant genera. Ascomycota was the phylum showing a larger number of genera decreased by the presence of plants, while most plant-affected genera of Basidiomycota and Glomeromycota increased by the presence of plants and included a great proportion of symbiotrophic genera.

### Response of Bacterial Taxa

In our soils, Proteobacteria and Actinobacteria were the most abundant bacterial phyla, as seen in other studies in the Mediterranean and other low organic matter soils (Curiel Yuste et al., 2014; Siles et al., 2014; Sun et al., 2016). Proteobacteria, a group of gram-negative bacteria that includes N_2_-fixing bacteria, decreased with mineral fertilization and increased with the application of compost. Indeed, 24 Proteobacteria genera increased by compost application including one BNF genera. The number of Proteobacteria genera increased by MF was much lower 3 as most genera 7 affected by mineral fertilization decreased (including the N-fixing *Microvirga* and *Burkholderia*) while *Devosia* increased.

The relative abundance of Proteobacteria did not relate with basal respiration or with the SIR of any of the tested substrates, irrespective of the plant–soil or mineral fertilization–soil context. Actinobacteria are a group of gram-positive bacteria responsible for the decomposition of organic matter and involved in humus formation processes (Kopecky et al., 2011) and are considered oligotrophic bacteria (Borrero et al., 2004; López-López et al., 2016; Avilés and Borrero, 2017). This phylum is highly abundant in compost (Trillas et al., 2006; Sui et al., 2019) and in forest soils (Zornoza et al., 2009). In the mineral fertilization experiment, the phylum Actinobacteria increased with mineral fertilization. The relative abundance of Actinobacteria coincided with decreased SIR for one out of four amino acids tested, two out of five sugars, and also one of the organic acids. This would suggest low metabolic activity for Actinobacteria in minerally fertilized soils. In agreement with our results, Actinobacteria abundance has been found to increase in conventionally managed soils in comparison with those organically managed (Li et al., 2012; Bonanomi et al., 2016), as well as after the addition of mineral fertilization (Fierer, 2017; Gu et al., 2019). This phylum increased with added mineral N, coinciding with lower microbial activities (Ramirez et al., 2012). The analyses of the most abundant genera showed that five Actinobacteria genera affected by mineral fertilization, including three N-fixing genera (*Frankia*, *Arthrobacter*, and *Streptomyces)*, increased in minerally fertilized soils.

The following group in abundance, Firmicutes, was shown to be the only one that responded to both mineral and organic fertilization, in agreement with Cesarano et al. (2017). Other authors have found increases of this phylum in organically managed soils (Bonanomi et al., 2016). This phylum did not relate to SIR in any of the studied cases. Increases of relative abundances of Firmicutes and Actinobacteria, as a consequence of adding N, did not show any positive effect on soil metabolic activities. This has been already pointed out by other authors (Ramirez et al., 2012) and indicates a functional shift toward less metabolically active microbial communities after adding N.

N-fixing bacteria were distributed in two phyla (Proteobacteria and Actinobacteria). Most N-fixing Protebacteria only increased in the presence of plants (*Bradyrhizobium*, *Rhizobium*, *Microvirga*, *Azoarcus*, and *Burkholderia*), while other Proteobacteria genera increased either with organic (*Methylobacterium*) or mineral fertilization (*Devosia*). *Methylobacterium* uses simple organic compounds as sources of energy (Jinal et al., 2020) and can live in the soil, in the rhizosphere, and in nodules or in other parts of plants (Andrews and Andrews, 2017). As in our soil, *Methylobacterium* relative abundances responded to organic fertilization and were not affected by the presence of plant, and it is suggested that this genus may be free living in our soils. N-fixing Actinobacteria appeared to be less plant dependent than N-fixing Proteobacteria as they responded to several of the studied treatments. Indeed, *Frankia* responded to all treatments: organic and mineral fertilization and to the presence of plants. *Micromospora* increased with organic fertilization and presence of plants, and *Arthrobacter* with mineral fertilization and presence of plants. N-fixing Actinobacteria can fix N as free-living bacteria in the rhizosphere (Rilling et al., 2018) or in some cases, as the case of *Frankia*, in symbiotic associations with actinorhizal plants (Sellstedt and Richau, 2013; Ardley and Sprent, 2021). As olive trees are not considered actinorhizal plants, we suggest *Frankia* in our soils were likely living in the rhizosphere. Some of these genera increased by fertilization practices or by plants (*Frankia* and *Methylobacterium*) can be classified as plant growth-promoting organisms or biocontrol agents (Papasotiriou et al., 2013; Jinal et al., 2020; Marappa et al., 2020).

The relative abundance of Bacteroidetes was greatly increased after adding both tested composts, while Chloroflexi mostly increased in SC1. In contrast, the relative abundances of Thaumarchaeota and Planctomycetes decreased in both SC1 and SC2 soils. In other studies, the relative abundance of Bacteroidetes also increased with compost and manure amendments (Fracchia et al., 2006; Danon et al., 2008; Maeda et al., 2011; Neher et al., 2013) and has been found to increase in soils restored with organic amendments (Bastida et al., 2009). In contrast, Chloroflexi and Planctomycetes decreased with compost amendments (Tian et al., 2015). In our study, the abundance of Chloroflexi increased in SC1 soils and showed a positive relationship with induced respiration by labile C- and N-rich C substrates.

Proteobacteria and Bacteroidetes decreased with mineral fertilization when compared with organic fertilization, similar to the findings of Gu et al. (2019). Indeed, our results show a low number of Proteobacteria and Bacteroidetes genera 3 increasing their relative abundance when minerally fertilized, while this figure was much higher 27 in compost-amended soils. Indeed, all Bacteroidetes genera affected by compost showed increases in their relative abundance. Bacteroidetes are believed to utilize relatively labile forms of C and exhibited copiotrophic attributes (Fierer et al., 2007; Ramirez-Villanueva et al., 2015). The relative abundance of Bacteroidetes showed the largest number of positive correlations with SIR, suggesting that the abundance of this phylum is promoted by adding compost and is therefore highly related to soil metabolism. In minerally fertilized soils, this phylum positively correlated with N-rich labile C substrates (lysine, L-alanine, and *N*-acetylglucosamine) as well as with sugars (trehalose) and aliphatic compounds (citric and oxalic acids). Thus, we suggest that Bacteroidetes’ metabolic activity responded positively to the addition of labile C and to N-rich labile substrates. This implies that this phylum may contribute to soil N mining mainly in non-minerally fertilized soils. Large effects of N amendments on the phylum Bacteroidetes have been found by other authors (Zhang et al., 2019). This phylum has also been reported to be capable of degrading cellulose and other complex organic compounds such as glucosinolates (Babin et al., 2019). In our data set, Bacteroidetes is the only bacterial phylum whose abundance showed a positive relationship with the induced respiration of a phenolic compound (orcinol).

Thaumarchaeota has been shown to participate dominantly and widely in soil N metabolic processes by increasing organic N metabolism with the addition of mineral N and urea (Li et al., 2019). These authors found increases of Thaumarchaeota after 50 years of adding mineral N and P fertilizers. In our experiment, the addition of the N-poor C1 reduced the abundance of Thaumarchaeota, while in these soils, it increased with mineral fertilization. In addition, Thaumarchaeota was the only phylum showing positive correlations with the addition of arginine, the richest N substrate that can be considered a storage form of organic N (Winter et al., 2015), suggesting that this phylum can metabolize N-rich organic compounds.

### Response of Fungal Taxa

In our soils, Ascomycota and Basidiomycota followed by Chytridiomycota and Glomeromycota were the most abundant fungal phyla, in agreement with other soils of contrasting climates and crops (Siles et al., 2014; Sadet-Bourgeteau et al., 2019). Composts showed a much lower number of fungal species and diversity than soils. Fungal phyla in composts are mainly composed of Ascomycota and Basidiomycota in low animal manure olive husk compost (C1) and Ascomycota in high animal manure olive husk compost (C2). Other studies on composting animal manure (Wang et al., 2018) also obtained high relative abundances of Ascomycota after 60 days of composting process, similar to our profile in C2. On the other hand, using composts based on plant material, both Ascomycota and Basidiomycota are reported to be the most dominant fungal phyla in composts. These two phyla are believed to be dominant in many composts due to their capacity to resist extreme conditions (high temperature, stress, nutrient deficiency) during the composting process and their capability to use multiple carbon sources (including lignocellulose polymers) (Yu et al., 2015).

Amending soils with C1 largely reduced the relative abundance of Ascomycota and increased the abundance of Basidiomycota, thus reducing fungal diversity. In contrast, C2-amended soils increased the relative abundance of Ascomycota with no changes in the abundance of Basidiomycota and general fungal diversity. Other authors have found large increases in Ascomycota when amending with N-rich compost (Abujabhah et al., 2016), while Ascomycota has been observed to decrease in rhizospheric soils in continuous cropping (Gao et al., 2019). In agreement with these latter authors, our results also showed a reduction of Ascomycota in the presence of olive plants in unamended soils and in soils amended with C1. The analyses of the most abundant genera showed that 26 genera of Ascomycota decreased by adding compost. This included eight saprotrophs, one pathotroph–symbiotroph (*Podospora*), and two symbiomtroph genera (*Hynobolites* and *Sarea*). Moreover, the presence of plants reduced six genera of Ascomycota within different combinations of pathotroph and saprotroph trophic modes. Four of these genera with pathotrophic character were *Acremonium*, *Fusarium*, *Verticillium*, and *Macrophomina*, while pure saprotrophic genera were *Ascobolus* and *Lomentospora*. It appears that the presence of plants reduced the relative abundance of pathogens. Similarly, adding compost reduced *Fusarium* and three other pathotrophic genera (*Alternaria*, *Coniothyrium*, *Ilyonectria*). In contrast, two pathotrophic Basidiomycota genera (*Pleurotus* and *Trichosporon*) were increased by adding compost.

The relative abundance of the phylum Basidiomycota increased in the presence of plants except in soils amended with C1. Plants increased seven Basidiomycota genera including three symbiotrophs (*Hydnum*, *Serendepita*, and *Inocybe*). *Hydnum* was also increased by the addition of compost. Chytridiomycota is a fungal phylum that is highly abundant in the rhizosphere and in certain agricultural soils (Liu et al., 2016; Tan et al., 2017). In spite of this, in our study, the phylum Chytridiomycota increased in rhizospheric soils, no genera of Chytridiomycota were increased by plants, and only one genus of this phylum was increased by compost addition (*Nowakowskiella*). Other studies have also shown increases in Chytridiomycota after cow manure vermicompost application (Zhao et al., 2017).

The SU from an olive tree orchard soil was the main source of the phylum Glomeromycota. The presence of Glomeromycota genera in composts was always found to be below 0.05% of relative abundance. According to Stürmer (2012), Glomeromycota is a monophyletic group containing AMF. This phylum increased in the presence of olive plants in all soils, and this increase affected the genera *Rhizophagus*, *Glomus*, and *Gigaspora*. In our study, amending soils with compost largely reduced this phylum in rhizospheric soils although it increased the genus *Acaulospora* and no effect was observed for mineral fertilization. *Acaulospora* sp. is inoculated in olive tree seedlings as AMF (Costa and Melloni, 2019). Other authors have found increases in AMF diversity in low-input agriculture and attributed such increases to crop rotations rather than to organic fertilization (Hijri et al., 2006). Indeed, Cavagnaro (2014) studying soils amended with high levels of green waste compost found decreased levels of AMF colonization and attributed this finding to high P levels in amended soils. Increases in Glomeromycota due to low nutrient availability have been also reported (Leff et al., 2015). In our study, the reduction of Glomeromycota was likely not related to the availability of nutrients or P, as adding large amounts of mineral nutrients including P did not have any significant effect.

Glomeromycota is the most abundant phylum of AMF in olive trees (Calvente et al., 2004). As expected, this phylum was independent of C sources used in SIR because most genera of this phylum are supposed to be directly fed by plants. This contrasts with the other fungal phyla that significantly correlated to different carbon sources. Ascomycota showed mainly negative correlations with several tested C substrates, and the plant–soil effect experiment showed a negative correlation with basal respiration. This contrasted with Basidiomycota and Chytridiomycota which showed positive correlations with basal respiration and with SIR for many of the tested substrates. Negative correlations with Ascomycota in the plant–soil experiment included C sources of different recalcitrance such as mono- and disaccharides, a non-protein amino acid (γ-aminobutyric acid), and even with a phenolic product (orcinol). This may be an indication that the species pertaining to this phylum, present in our soils, do not largely contribute to soil C and N metabolism. As in other studies (Sun et al., 2019), the relative abundance of Ascomycota showed an opposite trend compared with Basidiomycota. Basidiomycota increased with the presence of plants in two of the studied soils and appeared to metabolize C sources of contrasting N richness, such as the disaccharides trehalose or D-fructose and the N -rich compounds γ-aminobutyric acid and *N*-acetylglucosamine. Links between soil N, microbial N levels, and the abundance of Basidiomycota have been found in other studies (Sun et al., 2019). In addition, our study showed that the less abundant phylum, Chitridiomycota, showed a similar pattern for SIR in both experiments suggesting that both Basidiomycota and Chytridiomicota contributed to soil C and N metabolism. It has been reported that L-arabinose is degraded in early stages while D-galactose—a plant-specific sugar—accumulates at later stages of humification (Koivula and Hänninen, 2001). In our study, D-galactose-induced respiration increased with the presence of plants and positively correlated with both Basidiomycota and Chytridiomycota, thus suggesting that these fungal phyla responded to the addition of monosaccharides accumulating at later stages of humification. In contrast, SIR of the readily degradable sugar L-arabinose strongly correlated with the presence of Chytridiomycota and increased with the presence of plants in soils amended with compost. This strong correlation between Chytridiomycota relative abundance and basal respiration suggests that this phylum may contribute to soil microbial activity.

## Conclusion

The presence of olive sapling increased both bacterial and fungal richness and diversity and affected microbial function by increasing mean SIR. Olive saplings increased the relative abundance of five N-fixing genera of Proteobacteria *(Bradyrhizobium, Rhizobium, Microvirga, Azoarcus, and Burkholderia)*, three genera of Actinobacteria *(Arthrobacter, Frankia, and Micromonospora)*, and six symbiotroph fungal genera *(Rhizophagus, Glomus, Gigaspora, Serendipita, Inocybe, and Hydnum)*, while it reduced four pathotrophic genera *(Acremonium, Macrophomina, Fusarium, and Verticillium)*. Organic amendments affected a greater number of bacterial and fungal phyla and genera than plants. Organic amendments increased bacterial richness, while they decreased fungal richness in C1 and lowered fungal diversity in soils amended with both composts. Only one N-fixing genera was increased by organic amendments *(Methylobacterium)*, while mineral fertilization increased the relative abundance of three N_2_-fixing genera of Actinobacteria *(Frankia, Arthrobacter, and Streptomyces).* Frankia was the only N_2_-fixing genus that increased with the presence of plants and with organic and mineral fertilization. Finally, four pathotrophic fungal genera were decreased by organic amendments *(Alternaria, Coniothyrium, Fusarium, and Ilyonectria)*. The phylum Glomeromycota and several symbiotroph genera of Ascomycota, Basidiomycota, and Glomeromycota phyla decreased when adding composts in the presence of plants and were not affected by mineral fertilization. However, *Acaulospora* and *Hydnum* increased in organic-amended soils. Increases of SIR in organically amended soils were only observed when adding low N C1 compost and for a small number of substrates (arabinose, L-alanine, and γ-aminobutyric acid). Mineral fertilization increased the less metabolically active bacterial phyla (Actinobacteria and Firmicutes), while it reduced the most metabolically active phylum Bacteroidetes. Moreover, mineral fertilization increased bacterial diversity in soils amended with low N C1 compost. Somewhat surprisingly, three N-fixing Actinobacteria *(Arthrobacter, Frankia, and Streptomyces)* and one Proteobacteria *(Devosia)* increased with mineral fertilization.

*Basal respiration positively correlated with the relative abundance of two fungal phyla* (Basidiomycota and Chytridiomycota), while it did not relate with any bacterial phyla. The abundance of some bacterial (Bacteroidetes, Chloroflexi) and fungal (Basidiomycota, Chytridiomycota) phyla contributed to the metabolism of substrates of contrasted lability and N richness. The addition of compost favored the abundance of Bacteroidetes. The contribution of this phylum to soil metabolism was greater in minerally fertilized soils. This study provides new insight on the effects of olive saplings on soil bacterial and fungal microbiome under different nutrition programs: low and high N olive mill waste composts and mineral fertilization.

## Data Availability Statement

The datasets presented in this study can be found in online repositories. The names of the repository/repositories and accession number(s) can be found in the article/ Supplementary Material.

## Author Contributions

MT and JR got the funding for the research, designed the research, and set up the greenhouse experiment. MS-A processed the soil for DNA analyses. ML performed the SIR analyses. ML and GS analyzed the data. ML, GS, MS-A, MT, and JR wrote the manuscript. All authors contributed to the article and approved the submitted version.

## Conflict of Interest

The authors declare that the research was conducted in the absence of any commercial or financial relationships that could be construed as a potential conflict of interest.

## Footnotes

1 www.mrdnalab.com

2 http://rdp.cme.msu.edu

3 www.ncbi.nlm.nih.gov

## Supplementary Material

Data Sheet 1 Mineral fertilization, compost and plant affected microbiota (bacteria and fungi)

Data Sheet 2 Bacterial genera, counts and percentages.

Data Sheet 3 Fungal genera, counts and percentages.

Data Sheet 4 Bacterial phyla, counts and percentages.

Data Sheet 5 Fungal phyla, counts and percentages.
